# Luck in Old Shoes

**Published:** 1892-02

**Authors:** 


### LUCK IN OLD SHOES.

The Chinese value a pair of boots which have been worn by an upright magistrate, and the custom of wishing a friend a "happy foot" is still observed all through Europe. The casual putting on the left shoe on the right foot, pulling it on uneven or cross-wise, bursting the shoe latch or tie, lacing it wrong, and losing a button, are all bad signs.

A Yorkshireman will spit in his right shoe before putting it on, when going out on important business, to bring luck, and many an English girl has been known to hang her boots out of the window on St. Valentine's night for love-luck.

Professor Black tells of a singular superstition existing in England, which insists that if the youngest daughter of a family marries first her sisters must dance at her wedding without shoes so as to insure husbands for themselves.

Old-shoe throwing is done for many purposes. In Ireland the election of a person to almost any office is concluded by throwing an old shoe over his head.

The Gypsies say:

"Hurle after an old shoe,
I'll be merry what here I doe."

On the Isle of Man an old shoe is always thrown after the bride, as well as the groom when leaving their homes, and in the South the oldest person on the plantation, white or black, always throws an old shoe after any one starting on a long journey.

It is said that Mme. Patti and other women of high standing on the stage preserve most carefully the boots they wore at their debut, which they consider lucky to wear on the first night of engagements forever after.

